# Successful use of laparoscopic myomectomy to remove a giant uterine myoma: a case report

**DOI:** 10.1186/s13256-015-0771-9

**Published:** 2015-12-17

**Authors:** Huseyin Aksoy, Turgut Aydin, Özkan Özdamar, Özge Idem Karadag, Ulku Aksoy

**Affiliations:** 1 Department of Obstetrics and Gynecology, Kayseri Military Hospital, Kayseri, Turkey; 2 Department of Obstetrics and Gynecology, Kayseri Acıbadem Hospital, Kayseri, Turkey; 3 Department of Obstetrics and Gynecology, Kayseri Memorial Hospital, Kayseri, Turkey

**Keywords:** Giant myoma, Laparoscopic myomectomy

## Abstract

**Introduction:**

Uterine leiomyomas are the most common benign neoplasms of the female reproductive tract. Myomectomy is the preferred surgical treatment in reproductive-aged women who desire to retain their fertility. The use of a laparoscopic approach for large myomas is still controversial, although there are several compelling reasons for its use. The laparoscopic removal of giant uterine myomas is rare, and only a few cases have been published in the literature.

**Case presentation:**

We report the case of a 33-year-old white woman who was referred to our clinic with progressive abdominal distension. An ultrasonic examination revealed a markedly enlarged uterus containing a 17 cm uterine myoma. Laparoscopic myomectomy was selected as the treatment option. The laparoscopy confirmed the 17 cm fundal intramural myoma. The myoma was totally enucleated and removed without disturbing her endometrial cavity. The myometrial defect was repaired with a continuous suture using the V-loc suture in two layers. The entire myoma was removed using a tissue morcellator. The total weight of the myoma removed was 2005g, and the operation lasted for 140 minutes. Her postoperative course was unremarkable.

**Conclusions:**

Laparoscopic myomectomy offers many advantages compared with abdominal myomectomy. Although the use of a laparoscopic approach to treat very large myomas is controversial and technically demanding, we successfully performed a laparoscopic myomectomy in a patient with a giant myoma. This case confirms the efficiency, reliability, and safety of a minimally invasive surgical approach to treating a giant uterine myoma. Laparoscopic myomectomy can be performed by experienced surgeons regardless of the size of the myoma.

## Introduction

Uterine leiomyomas, originating from the uterine smooth muscle, are the most common benign neoplasms of the female reproductive tract. They are found in 25 to 30 % of women of reproductive age, as many as 50 % of women over the age of 35 years and approximately 70 % of women over the age of 50 years; the prevalence of uterine leiomyomas increases during reproductive age and decreases after menopause [1, 2]. Leiomyomas range in size from microscopic to bulky masses that can distort and enlarge the uterus. Although most of myomas are small and do not require treatment unless they cause symptoms, on rare occasions, myomas can grow extremely large [3]. For myomas requiring surgical treatment, usually a myomectomy or hysterectomy is performed, depending on the desire of the patient to remain fertile and the severity of the symptoms. A myomectomy is preferred for reproductive-aged women with symptomatic myomas who desire to maintain their fertility [4].

A laparoscopic myomectomy (LM) offers several advantages over the laparotomic technique, such as a shorter hospitalization, less postoperative pain, faster recovery, and lower risk of postoperative adhesions [5–7]. The published studies on LMs indicate that they are performed more frequently for small- and medium-sized myomas [8, 9]. The use of a laparoscopic approach for treating large myomas is controversial due to the increased difficultly of excision, cleavage removal and repair of the myometrial defect, the increased operative time, and the increased risk of perioperative bleeding and conversion to laparotomy compared with that of smaller myomas [5]. There are a few reports in the literature describing the laparoscopic removal of large myomas [10–12]. Here, we present the case of a 33-year-old woman with a giant uterine myoma that was successfully removed using a laparoscopic approach. In addition, we examine the case in terms of surgical technique and equipment and review the international literature.

## Case presentation

A 33-year-old, white, gravida 2, para 2 woman was referred to our Gynecology Department with intermittent abdominal pain that had intensified during the previous 3 months and progressive abdominal swelling during the previous 2 years. Her past medical and gynecologic history was otherwise unremarkable. No familial history of any disease was reported. A physical examination revealed a firm giant palpable abdominal mass with identifiable borders. The mass extended to her umbilicus and measured 15 cm above her symphysis pubis. These findings were confirmed by abdominal sonography. The abdominal ultrasonic examination revealed a markedly enlarged and lobular uterus containing intramural uterine leiomyomas, the largest measuring 17×15 cm without ascites in her abdominal cavity. No additional pathology was noted in the remainder of her pelvis or abdomen. The results of the routine laboratory testing, including a complete blood count, serum electrolyte levels and biochemical tests, were within normal limits. On the basis of these findings, a giant intramural myoma was assumed, and myomectomy was selected as the treatment.

**Fig. 1:**
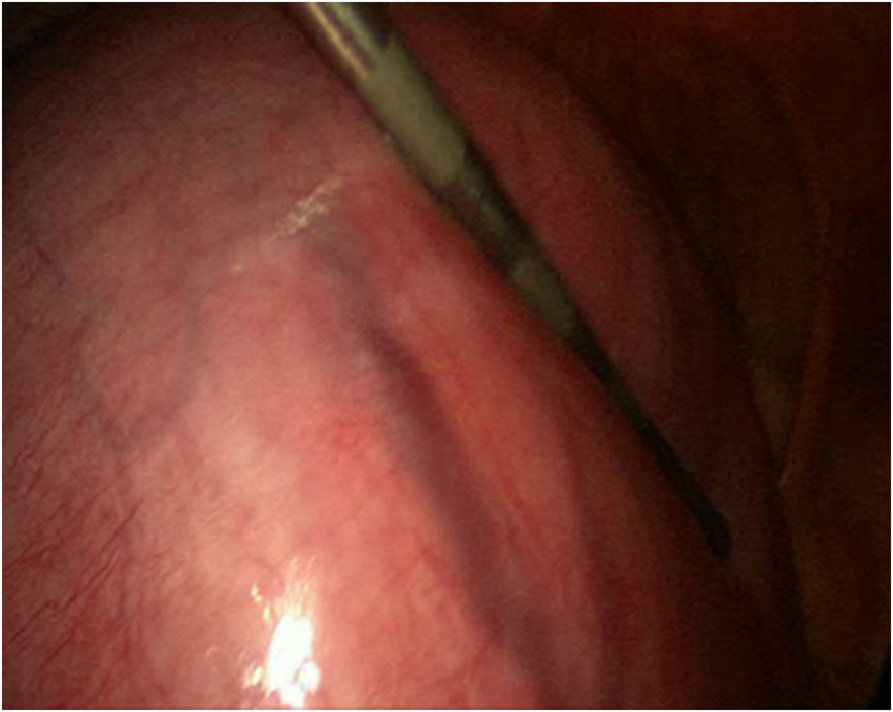
Laparoscopic view of uterine myoma. The whole pelvic cavity is filled by the uterine myoma with a maximum diameter of 17 cm

**Fig. 2:**
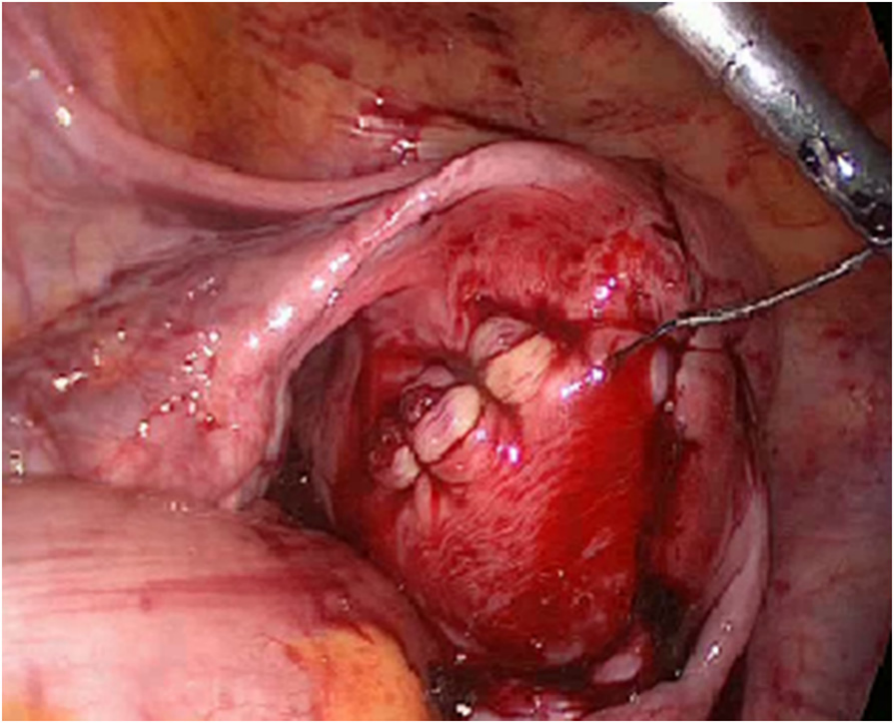
After removal of the giant myoma, the myometrial defect was repaired using V-loc (Covidien UK; unidirectional barbed) continuous self-retaining suture in two layers

She was offered laparoscopic removal of the myoma. Pneumoperitoneum was achieved using a supraumbilical Veress needle until an intra-abdominal pressure of 12 mmHg was reached. We first placed a midline supraumbilical 10 mm port for the telescope, and then two 5 mm accessory trocars were positioned in the left and right lateral quadrants visualized via a 10 mm telescope inserted through the supraumbilical port. The left and right accessory ports were inserted lateral to her deep inferior epigastric arteries and higher than usual; the accessory trocars were inserted sufficiently high enough to provide an unobstructed passage to the myomas for the laparoscopic instruments. Intra-abdominal visualization revealed an enlarged, lobular uterus containing a fundal intramural myoma with a maximum diameter of 17 cm, as diagnosed by ultrasound (Fig. 1). The adnexa on both sides, round ligaments, and other pelvic and abdominal organs were normal. A myomectomy was performed using the standard technique as described elsewhere [13]. A vertical incision was made on the prominent part of the principal myoma using a monopolar hook. The cleavage plane between the myoma and its surrounding connective tissues was then dissected. When the myoma was identified, the myoma was fixed, and enucleation was then accomplished by traction on the myoma with a tenaculum clamp, associated with countertraction on the uterus-facilitated dissection. The myoma was completely enucleated and removed without disturbing the endometrial cavity. A Harmonic ultrasonic scalpel (Ethicon Endo-Surgery Inc, Cincinnati, OH, USA) was used for most of the procedure. Bipolar coagulation was used when extra hemostasis was required. The myometrial defect and edges were closed with a continuous suture using a V-loc unidirectional barbed suture (Covidien, UK) in two layers (Fig.2). The left accessory 5 mm port was converted to a 10 mm one for the insertion of the morcellator. The entire myoma was removed using an electromechanical Rotocut G1 tissue morcellator (Karl Storz, Tuttlingen, Germany). The total intraoperative blood loss was 720 mL, the total weight of the myoma removed was 2005 g and the operation lasted for 140 minutes.

There were no major intraoperative complications. The final histopathological examination confirmed the diagnosis of a uterine leiomyoma. The postoperative course was unremarkable, and the patient was discharged on the second postoperative day.

## Discussion

A literature search indicated that the use of a LM to remove of a leiomyoma of this size, with a maximum diameter of 17 cm (2005 g), is very rare. However, the entire myoma was successfully removed laparoscopically. Despite this success, the use of a laparoscopic approach to treat large myomas is still controversial and represents a significant surgical challenge. The difficulties of cleavage, removal and repair of the myometrial defect and the increased operative time and risk of perioperative bleeding and conversion to laparotomy are the major concerns regarding the use of a LM to treat large myomas [5]. Due to these compelling factors, the surgical treatment options and approaches are not standardized, and the appropriate management of patients with very large myomas is complex and requires exceptional skill.

Uterine leiomyomas are benign tumors that arise from the overgrowth of smooth muscle and connective tissue in the myometrium and are the most common solid benign neoplasm of the female genital tract. The size of leiomyomas varies from microscopic to lesions of considerable size. Although most myomas are asymptomatic and usually small in size, they can reach >10 cm in size [3]. Most myomas do not require intervention unless they cause symptoms. For symptomatic myomas, hysterectomy offers a definitive solution. However, it is not the preferred solution for women of reproductive age who desire to maintain their fertility or those who want to preserve their uterus for cultural, social, or emotional reasons. Myomectomy remains the treatment of choice and gold standard for these patients [4]. LM offers many advantages compared with an abdominal myomectomy due to its minimally invasive nature and potentially a woman’s fertility can be retained [5–7]. However, the use of a laparoscopic approach to treat very large myomas is still controversial and technically very demanding [5].

The laparoscopic removal of giant uterine myomas is rare, and only a few cases have been published in the literature [10–12]. The largest uterine myoma removed laparoscopically measured 21 cm and weighed 3400 g [10]. In 2003, Sinha *et al.* conducted a prospective study to evaluate the feasibility, complications, and conversion rate of laparoscopic excision of very large myomas [10]. The authors included 51 women with at least one myoma larger than 9 cm, and they removed 78 myomas laparoscopically in these 51 patients. The largest myoma removed was 21 cm in their prospective study. The authors reported that LM is a safe alternative to laparotomy for very large myomas. A similar study conducted by Yoon *et al.* demonstrated that a laparoscopic approach is an efficient and feasible myomectomy method to treat large myomas [11]. In this prospective study, 51 patients with myomas 8 cm or larger in diameter who underwent LM were investigated over a 3-year period. The largest myoma successfully removed in the study was 15.2 cm. In a case report in 2013, a 34-year-old infertile woman underwent diagnostic laparoscopy because of a large abdominal mass, and an 18 cm myoma was laparoscopically removed [12]. In our case, the maximum diameter of the myoma was 17 cm, and the entire myoma was successfully removed laparoscopically without disturbing the endometrial cavity.

On 24 November 2014, the US Food and Drug Administration (FDA) issued a statement warning against using laparoscopic power morcellators in women undergoing hysterectomy or myomectomy for uterine fibroids [13]. One of the major concerns over morcellation of an occult cancer is delayed diagnosis because of misinterpretation of the initial pathologic specimen [14]. Another major concern over morcellation of an occult malignancy is the possibility of the seeding of cancer throughout the peritoneal cavity. In the recent literature there exist retrospective cohort studies, which described up-staging of sarcoma secondary to peritoneal spread after morcellation [15, 16]. Minimally invasive gynecologic surgeons who perform laparoscopic intraperitoneal morcellation should be aware of the recent FDA warning and litigation arising from use of morcellation devices with claims of intraperitoneal dissemination of cancerous cells.

Strategies to continue to allow surgeons to provide minimally invasive surgery to patients while minimizing the risk of the spread of occult malignancy involve refinement of contained morcellation techniques. There are reports of power morcellation within an endoscopic bag [17, 18], extracorporeal morcellation [19, 20], and transvaginal insertion of the anchor tissue retrieval system [21]. New surgical methods are being investigated so that women with large uterine leiomyomata can still be offered laparoscopic surgery.

## Conclusions

This case confirms the efficiency, reliability, and safety of a minimally invasive surgical approach to removing a giant uterine myoma. Thus, LM can be considered an alternative to the traditional abdominal myomectomy in patients with large myomas. Although a laparoscopic approach for large myomas has several challenges, it does represent a convenient option for the minimally invasive removal of very large myomas in the hands of an expert surgeon with appropriate surgical equipment.

## Consent

Written informed consent was obtained from the patient for publication of this case report and accompanying images. A copy of the written consent is available for review by the Editor-in-Chief of this journal.

## Abbreviations

FDA: Food and Drug Administration
LM: laparoscopic myomectomy
